# Psychological inflexibility and insomnia as serial mediators of the link between social media addiction and romantic relationship quality

**DOI:** 10.3389/fpsyg.2026.1847722

**Published:** 2026-07-27

**Authors:** Saide Umut Zeybek Çakti

**Affiliations:** Faculty of Education, Department of Guidance and Psychological Counseling, Mugla Sitki Koçman University, Mugla, Türkiye

**Keywords:** insomnia, psychological inflexibility, romantic relationship quality, serial mediation model, social media addiction, structural equation modeling

## Abstract

Widespread social media use may be one of the most important factors influencing individuals' romantic relationships. In particular, excessive, uncontrolled, and problematic social media use may negatively affect both individuals' psychological processes and their relational functioning in interpersonal interactions. The aim of the present study is to examine the serial predicting roles of psychological inflexibility and insomnia in the relationship between social media addiction and romantic relationship quality. The sample consisted of 466 adults from different regions of Türkiye. Of the participants, 368 were female (79%) and 98 were male (21%). The mean age of the participants, whose ages ranged from 18 to 72, was 34.32 years (*SD* = 10.93). In line with the aim of the study, the relationships among variables were examined using correlation analysis, and structural equation modeling was employed to test the mediation model. The findings indicated that social media addiction was positively associated with psychological inflexibility and insomnia and negatively associated with romantic relationship quality. According to the structural equation modeling results, the model fit indices suggested that the proposed model demonstrated a good fit to the data. The analysis further revealed that psychological inflexibility and insomnia played serial predicting roles in the relationship between social media addiction and romantic relationship quality. These findings suggest that social media addiction may increase levels of psychological inflexibility, which in turn may contribute to sleep-related problems, and that these processes may be reflected in lower perceived romantic relationship quality. Accordingly, interventions aimed at enhancing psychological flexibility and improving sleep patterns may be useful in understanding the relational consequences of social media addiction.

## 1 Introduction

In recent years, with the rapid acceleration of digitalization, individuals' daily routines and patterns of interpersonal interaction have undergone a significant transformation. The widespread use of digital technologies, particularly social media platforms, has substantially reshaped the ways in which individuals engage in interpersonal interactions. Although social media serves as a tool that facilitates communication and strengthens social connections among individuals, its excessive, unregulated, and problematic use has been associated with various psychological and relational difficulties (Andreassen, 2015; Kuss and Griffiths, 2017). In this context, social media may function as a means of enhancing social capital while also being considered a contextual factor that may pose risks to individuals' emotional and cognitive processes. Growing evidence suggests that problematic social media use may undermine individuals' psychological wellbeing and may be associated with a range of negative outcomes in interpersonal relationships (Huang, 2022). More recently, researchers have argued that the consequences of addictive behaviors extend beyond individual psychological and physical health to interpersonal and romantic domains. For example, Oltra et al. (2024) demonstrated that tobacco use negatively influenced partner selection and reduced individuals' willingness to establish stable romantic relationships. These findings suggest that addictive behaviors may shape not only individual wellbeing but also the formation and maintenance of close romantic relationships. Accordingly, examining the relational consequences of problematic social media use represents an important extension of the broader addiction literature. Within this broader framework, it becomes crucial to move beyond individual-level factors of social media use and examine its associations with close interpersonal relationships, particularly romantic relationship functioning.

Within the context of romantic relationships, social media use has emerged as an important factor that reshapes interactions between partners. Recent research has continued to demonstrate that problematic technology use, including excessive social media engagement, may undermine relationship functioning by increasing conflict, reducing emotional responsiveness, and disrupting interpersonal communication (Karaman and Arslan, 2024; Ni et al., 2025). Collectively, these findings indicate that digital behaviors have become an increasingly important determinant of romantic relationship quality. In particular, interactions occurring through social media have been shown to increase feelings of jealousy and insecurity between partners, which may lead to lower relationship satisfaction (Muise et al., 2009). Similarly, social media use has been associated with increased surveillance behaviors in romantic relationships, which may be linked to relationship dissatisfaction (Elphinston and Noller, 2011). Studies also report that social media use may be associated with increased conflict, heightened jealousy, and decreased relationship satisfaction in romantic relationships (Fox and Moreland, 2015; Muise et al., 2009). These findings suggest that social media interactions may not only alter communication patterns but also reshape the emotional climate and trust dynamics within relationships. In addition, increased social media use may reduce face-to-face interaction and weaken emotional intimacy, which may be reflected in lower relationship quality. Accordingly, social media may exhibit a dual role in romantic relationships, functioning as both a bonding and a weakening factor. Taken together, these findings suggest that social media addiction may be an important variable associated with perceived romantic relationship quality.

To better understand the relationship between social media addiction and romantic relationship quality, it is important to examine the underlying mechanisms of this association. Such relationships may be better conceptualized as multidimensional and process-based rather than operating through predictive links. A review of relevant literature suggests that this association may not be limited to a single pathway but instead operate through predictive pathways involving individuals' psychological and physiological processes (Fox and Moreland, 2015; Roberts and David, 2016). In this context, psychological inflexibility and insomnia may be considered as two key variables with the potential to explain the relationship between social media addiction and romantic relationship quality. Examining these variables together may provide a more comprehensive and in-depth perspective on understanding the relational outcomes of social media addiction.

Beyond its theoretical contribution, examining these mechanisms has important applied relevance. From a preventative perspective, identifying modifiable psychological and behavioral processes may support early interventions designed to reduce the relational consequences of problematic social media use. From a clinical and counseling perspective, understanding the roles of psychological inflexibility and insomnia may help practitioners develop more comprehensive interventions for individuals and couples experiencing technology-related relationship difficulties. Furthermore, these findings may contribute to the growing field of digital wellbeing by emphasizing that the consequences of problematic social media use extend beyond individual mental health to the quality of close romantic relationships.

### 1.1 Psychological inflexibility and insomnia as mediators

The growing interest in the relationship between social media addiction and romantic relationship quality highlights the importance of examining the underlying mechanisms of this association. Considering how social media use predicts individuals' psychological functioning and biopsychosocial processes, this relationship may extend beyond a predictive link (Fox and Moreland, 2015; Kuss and Griffiths, 2017). This suggests that social media use may be better understood through multi-layered and interactive processes. In this context, examining the psychological and behavioral pathways through which social media addiction may influence relationship quality presents a significant research need. Accordingly, investigating this relationship through predicting mechanisms may contribute to addressing a meaningful gap in the literature.

To explain the association between social media addiction and romantic relationship quality, it may be important to examine individuals' internal psychological processes. In this context, psychological inflexibility is defined as the tendency to avoid internal experiences and the inability to regulate behavior in accordance with one's values in a flexible manner (Hayes et al., 2006). From the perspective of Acceptance and Commitment Therapy, psychological inflexibility is characterized by difficulties in relating flexibly to present-moment experiences due to processes such as experiential avoidance and cognitive fusion. Individuals with higher levels of psychological inflexibility may be more likely to experience difficulties in interpersonal relationships (Kashdan and Rottenberg, 2010). These individuals may have difficulty regulating their emotional responses and may display more rigid, defensive, and avoidant patterns of interaction within relationships. In this regard, social media addiction may be associated with romantic relationship quality through psychological inflexibility. When social media is used as a means of avoiding negative internal experiences, it may reinforce psychological inflexibility.

In addition, social media use, particularly during night-time hours, may disrupt individuals' sleep patterns and contribute to increased symptoms of insomnia (Levenson et al., 2016). Exposure to screens, characterized by blue light emissions and heightened cognitive arousal, negatively predicts the ability to initiate sleep and overall sleep quality (Chang et al., 2015). Insomnia has been associated with difficulties in emotional regulation and impairments in interpersonal functioning (Troxel et al., 2007). Insufficient sleep may lead individuals to experience emotional responses more intensely and less controllably, which may be reflected in increased conflict and communication problems within romantic relationships (Gordon and Chen, 2014). In this context, insomnia may be considered an important pathway linking social media addiction to romantic relationship quality. Additionally, the chronic nature of sleep disturbances negatively predicts individuals' overall psychological functioning and shows a predictive link with lower relational satisfaction via these psychological pathways.

Drawing on the research and theoretical framework, social media addiction may be associated with increased psychological inflexibility which may, in turn, contribute to the emergence of sleep-related problems. In other words, a rigid and avoidant stance toward internal experiences may influence individuals' behavioral patterns and lead to disruptions in sleep regulation. When considered together, these processes may be reflected in weakened interpersonal functioning and lower romantic relationship quality. Accordingly, it may be assumed that a sequential predictive framework links these variables. To date, no study has examined the combined and serial predicting roles of psychological inflexibility and insomnia in this relationship. In this respect, the present study aims to contribute to the literature by examining these variables within a comprehensive and integrative model. Based on this framework, the present study aims to investigate the serial predicting roles of psychological inflexibility and insomnia in the relationship between social media addiction and romantic relationship quality. By identifying potentially modifiable psychological and sleep-related processes, the study also seeks to provide evidence that may inform preventive strategies, psychological counseling interventions, and digital wellbeing initiatives aimed at promoting healthier romantic relationships. Accordingly, the following hypotheses were tested:

*H1*. Psychological inflexibility mediates the relationship between social media addiction and romantic relationship quality.

*H2*. Insomnia mediates the relationship between social media addiction and romantic relationship quality.

*H3*. Psychological inflexibility and insomnia serially mediate the relationship between social media addiction and romantic relationship quality.

## 2 Method

### 2.1 Participants and procedure

A total of 466 adults from different regions of Türkiye participated in the study. Of the participants, 368 were female (79%) and 98 were male (21%). The mean age of the participants was 34.32 years (*SD* = 10.93; age range = 18–72). Regarding relationship status, 58.4% of the participants were married (*n* = 272), while 41.6% were in a romantic relationship (*n* = 194). In terms of educational level, 41% of the participants held a bachelor's degree (*n* = 191), 34.1% had completed high school (*n* = 159), 18.5% had an associate degree (*n* = 86), 6.2% had a master's degree (*n* = 29), and 0.2% had a doctoral degree (*n* = 1). Detailed demographic characteristics of the participants are presented in Table 1.

**Table 1:** Participants' characteristics.

Variable	Frequency	%
Gender		
Female	368	79.0
Male	98	21.0
Marital status		
Married	272	58.4
In a relationship	194	41.6
Educational status		
High school	159	34.1
Associate degree	86	18.5
University	191	41.0
Master's	29	6.2
Ph.D.	1	0.2

Data collection was carried out online via Google Forms between September 10, 2025, and November 7, 2025. The online survey link was widely disseminated through the social media accounts (e.g., WhatsApp, Instagram, and LinkedIn) of the authors as well as their extended personal and professional networks, utilizing a convenience and snowball sampling approach to achieve a diverse outreach. To ensure strict procedural transparency and replicability, specific eligibility criteria and screening procedures were implemented. Since the study investigated romantic relationship quality, the inclusion criteria required participants to be at least 18 years of age and currently involved in an active romantic relationship or marriage. At the very beginning of the online survey, a mandatory screening question asked participants whether they were currently in an active romantic relationship or married. If a participant responded “No,” the survey automatically terminated for them, and they were redirected to the end of the form without access to the psychometric scales. To prevent duplicate responses and maintain strict data integrity, the “limit to 1 response” feature of Google Forms was enabled. This configuration strictly required participants to authenticate via their Google accounts and technically prohibited any subsequent submissions from alternative or secondary email addresses on the same session. Participation was entirely voluntary, and no financial compensation or rewards were provided. Digital informed consent was obtained from all participants prior to their formal involvement. Participants were clearly informed about the purpose of the study and explicitly reassured that they could withdraw from the study at any stage without any consequences.

### 2.2 Measures

#### 2.2.1 Bergen social media addiction scale

To assess social media addiction, the scale developed by Andreassen et al. (2016) and adapted into Turkish by Demirci (2019) was used in this study. This six-item measure is rated on a five-point Likert scale ranging from “*1* = *very rarely*” to “*5* = *very often*.” The items reflect the core components of addiction, and higher total scores indicate higher levels of social media addiction. The original version of the scale was reported to demonstrate good psychometric properties, with a high level of internal consistency (α = 0.88). In the present study, the Cronbach's alpha internal consistency coefficient of the scale was calculated as 0.73 (see Table 2).

**Table 2:** Descriptive statistics, reliability coefficients, and correlations.

Variables	Descriptive statistics and reliabilities						Correlations			
	Mean	SD	Skewness	Kurtosis	α	ω	1	2	3	4
1. Psychological inflexibility	26.62	9.37	−0.06	−0.40	0.82	0.82	–			
2. Relationship quality	32.59	7.12	−0.65	−0.16	0.84	0.85	−0.34^**^	–		
3. Social media addiction	17.35	5.19	0.04	−0.26	0.73	0.73	0.43^**^	−0.13^**^	–	
4. Insomnia	6.94	4.14	0.67	0.14	0.79	0.79	0.41^**^	−0.26^**^	0.33^**^	–

^**^p < 0.01.

#### 2.2.2 Perceived romantic relationship quality scale

To assess overall perceived romantic relationship quality, the short form of the scale developed by Fletcher et al. (2000) was used. The Turkish adaptation of the scale was developed by Sagkal and Özdemir (2018). This six items are rated on a seven-point Likert scale ranging from “*1* = *not at all*” to “*7* = *very much*.” The scale does not include any reverse-coded items, and higher total scores indicate higher levels of perceived romantic relationship quality. The original version of the scale was reported to demonstrate good psychometric properties, with an adequate level of internal consistency (α = 0.86). In the present study, the internal consistency coefficient of the scale was found to be 0.84 (see Table 2).

#### 2.2.3 Acceptance and action questionnaire-II

To assess levels of psychological inflexibility, the Acceptance and Action Questionnaire-II developed by Bond et al. (2011) and adapted into Turkish by Yavuz et al. (2016) was used. This seven-item self-report tool is rated on a seven-point Likert scale ranging from “*1* = *never true*” to “*7* = *always true*.” All items are negatively worded, and higher total scores indicate higher levels of psychological inflexibility. The Acceptance and Action Questionnaire-II is designed to assess experiential avoidance and cognitive rigidity, which are core components of psychological inflexibility, and it has been reported to demonstrate good psychometric properties. The original version of the scale has an adequate level of internal consistency (α = 0.84). In the present study, the Cronbach's alpha coefficient was calculated as 0.82 (see Table 2).

#### 2.2.4 Athens insomnia scale

To assess insomnia symptoms, the scale developed by Soldatos et al. (2000) and adapted into Turkish by Elbi et al. (2024) was used. This eight-item measure is rated on a four-point Likert scale ranging from “*0* = *no problem at all*” to “*3* = *very serious problem*.” Higher total scores indicate higher levels of insomnia symptoms. The Athens Insomnia Scale was developed to assess the core dimensions of insomnia, including difficulties in sleep initiation, sleep maintenance, early morning awakening, and daytime functioning, and it was reported to demonstrate good psychometric properties. The original version of the scale has a high level of internal consistency (α = 0.87). In the present study, the Cronbach's alpha coefficient was found to be 0.79.

### 2.3 Ethical statement

This study was approved by the Human Research Ethics Committee of [Omitted] University (Approval No. = [Omitted], Date = [Omitted]). Furthermore, all procedures were carried out in accordance with the ethical standards of the 1964 Helsinki Declaration and its later amendments.

### 2.4 Data analysis

Prior to the main analyses, preliminary analyses were conducted. In this context, descriptive statistics (means and standard deviations), normality assumptions, reliability analyses, and correlation analyses were examined to explore the relationships among variables. The assumption of normality was assessed based on skewness and kurtosis values, and the results indicated that the values fell within acceptable ranges. In addition, internal consistency coefficients were calculated for all scales to assess the reliability of the measurements.

Regarding the examination of the predicting roles of psychological inflexibility and insomnia in the relationship between social media addiction and romantic relationship quality, the two-step structural equation modeling approach was adopted, as proposed by (Anderson and Gerbing 1988). In this approach, the validity of the measurement model is tested first, followed by the evaluation of the hypothesized structural model. This approach enables the examination of the specific intervening paths linking the variables via psychological inflexibility (H1) and insomnia (H2), as well as the combined path sequence involving both variables (H3). All statistical analyses were conducted using IBM SPSS Statistics 22, AMOS 24, and JASP 0.18.3.0.

After confirming the measurement model, the hypothesized structural model was evaluated. In this framework, the predictive pathways from social media addiction to romantic relationship quality were examined through psychological inflexibility and insomnia. Model fit was evaluated using CFI, NFI, GFI, IFI, RMSEA, and SRMR. RMSEA and SRMR values below 0.08 and CFI, NFI, GFI, and IFI values above 0.90 were considered indicative of acceptable model fit (Hoyle and Panter, 1995). The significance of the intervening pathways was tested using a bootstrapping procedure with 5,000 resamples and 95% confidence intervals. According to Hayes (2018), an intervening sequence is considered statistically significant if the bootstrap confidence interval does not include zero.

To evaluate the adequacy of the sample size, recommendations for covariance-based structural equation modeling (SEM) were considered. Although an *a priori* power analysis was not conducted prior to data collection, the proposed model consisted of four latent variables represented by 12 observed indicators. According to Kline (2023), sample sizes exceeding 200 are generally considered adequate for SEM, while larger samples contribute to more stable parameter estimation. Therefore, the final sample of 466 participants exceeded commonly recommended thresholds and was considered sufficient to obtain stable parameter estimates and reliable model estimation.

The parceling technique is a functional method for reducing measurement errors that occur in unidimensional measuring instruments (Little et al., 2002). The literature indicates that parceling strengthens the assumptions of normality and reliability, particularly in instruments assessing personality traits (Nasser-Abu Alhija and Wisenbaker, 2006), and optimizes model–data fit by suppressing systematic errors produced by individual items (Yang et al., 2010). This technique, applied by grouping items into subsets, was preferred due to the unidimensional nature of the constructs in the current study.

The selection of this method was driven by its capacity to provide higher reliability, stronger communality, a reduced likelihood of distributional violations, and a higher ratio of common-to-unique factor variance (Little et al., 2013). Additionally, Little et al. (2013) emphasize that parceling offers an analytical advantage by reducing both sources of sampling error and the indicator-to-sample-size ratio.

## 3 Results

### 3.1 Preliminary analyses

Descriptive statistics for the variables examined in the study indicated that the mean score was 26.62 (*SD* = 9.37) for psychological inflexibility, 32.59 (*SD* = 7.12) for romantic relationship quality, 17.35 (*SD* = 5.19) for social media addiction, and 6.94 (*SD* = 4.14) for insomnia. An examination of the distribution of the study variables showed that skewness values ranged from −0.06 to 0.67 and kurtosis values ranged from −0.40 to 0.14. These values fall within the commonly accepted range of −1 to +1, suggesting that the data meet the assumption of normality (Tabachnick and Fidell, 2013). In addition, the reliability coefficients of the measurement instruments used in the study ranged from acceptable to good levels, indicating adequate internal consistency of the measures. The results of the correlation analysis further indicated that all variables included in the study were significantly associated with each other (see Table 2).

### 3.2 Statistical assumptions

The results of the study indicated that the skewness values ranged from −0.65 to 0.67 and the kurtosis values ranged from −0.40 to 0.14, suggesting that the data met the assumption of normality. In addition, all reliability coefficients were above 0.73, indicating acceptable internal consistency. The variance inflation factor (VIF) values ranged from 1.04 to 1.10, and the tolerance values ranged from 0.91 to 0.97, indicating that there were no serious multicollinearity problems among the variables. Furthermore, the Durbin–Watson value was 1.99, suggesting no autocorrelation problem in the residuals. The condition index values were below the critical threshold (Max CI = 14.64), and standardized residual values ranged from −2.50 to 2.80, indicating no serious multicollinearity or outlier problems. Accordingly, the findings suggested that the statistical assumptions required for the analyses were adequately met (Field, 2016). Finally, Common Method Bias (CMB) was examined using Harman's single-factor test. The results indicated that a single factor accounted for 34.80% of the total variance, remaining well below the commonly suggested threshold of 50%. Therefore, the findings suggest that common method variance does not pose a serious threat to the validity of the results.

### 3.3 Measurement model reliability and validity

Prior to testing the structural model, the reliability, convergent validity, and discriminant validity of the measurement model were evaluated (Table 3). Internal consistency reliability was assessed using Cronbach's alpha (α) and McDonald's omega (ω) coefficients. As recommended by Hair et al. (2019), coefficients above 0.70 indicate satisfactory reliability. The obtained α coefficients ranged from 0.728 to 0.837, while ω coefficients ranged from 0.763 to 0.869, demonstrating adequate internal consistency for all latent constructs.

**Table 3:** Measurement model reliability, convergent validity, and discriminant validity (HTMT) results.

Construct	α	ω	AVE	(1)	(2)	(3)	(4)
(1) Social media addiction	0.728	0.763	0.415	–			
(2) Insomnia	0.786	0.851	0.447	0.391	–		
(3) Romantic relationship quality	0.837	0.869	0.554	0.154	0.278	–	
(4) Psychological inflexibility	0.822	0.838	0.442	0.522	0.506	0.347	–

α, Cronbach's alpha; ω, McDonald's omega; AVE, average variance extracted.

Values below the diagonal represent the Heterotrait–Monotrait (HTMT) ratios.

Convergent validity was evaluated using the average variance extracted (AVE). The AVE value for romantic relationship quality (0.554) exceeded the recommended threshold of 0.50. Although the AVE values for social media addiction (0.415), insomnia (0.447), and psychological inflexibility (0.442) were slightly below the recommended threshold of 0.50, the corresponding reliability coefficients exceeded 0.60. Therefore, following the recommendation of (Fornell and Larcker 1981), convergent validity was considered acceptable.

Discriminant validity was assessed using the Heterotrait–Monotrait (HTMT) ratio of correlations. The HTMT values ranged from 0.154 to 0.522, all of which were well below the conservative cutoff value of 0.85 recommended by Henseler et al. (2015). These findings indicate satisfactory discriminant validity, suggesting that each latent construct represents a conceptually distinct phenomenon.

As shown in Table 3, the measurement model comprised four latent constructs—psychological inflexibility, insomnia, social media addiction, and romantic relationship quality—represented by 12 observed indicators. The measurement model demonstrated a good fit to the data, χ^2^(48, *N* = 466) = 83.199, *p* < 0.001, χ^2^/df = 1.73, RMSEA = 0.040, SRMR = 0.036, CFI = 0.984, NFI = 0.963, GFI = 0.972, IFI = 0.984, and TLI = 0.978. Furthermore, all standardized factor loadings were statistically significant and within acceptable ranges, indicating that the observed indicators adequately represented their respective latent constructs. Overall, these findings support the reliability and construct validity of the measurement model and indicate that it was appropriate for subsequent structural model analyses.

### 3.4 Structural model

**Figure 1:**
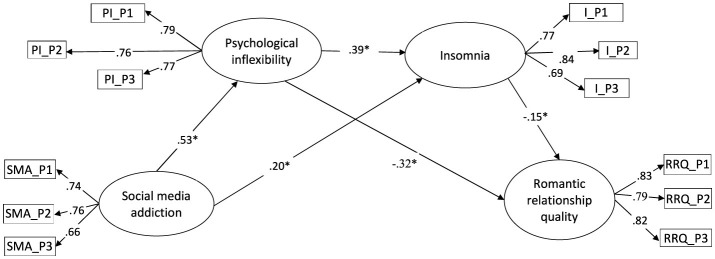
Structural equation modeling for the serial mediation model. * *p* < 0.05; SMA, parcels of social media addiction; PI, parcels of psychological inflexibility; I, parcels of insomnia; RRQ: parcels of romantic relationship quality.

In the first stage, a partial mediation framework including all paths was tested. However, the predictive link between social media addiction and romantic relationship quality was found to be statistically nonsignificant (β = 0.126, *p* > 0.05). Accordingly, this specific path coefficient was removed, and a full intervening model was tested. The fit indices for this predictive model indicated a good fit to the data: χ^2^ (49, *N* = 466) = 86.412, *p* < 0.001; χ^2^/df = 1.764; SRMR = 0.037; RMSEA = 0.041; GFI = 0.971; CFI = 0.983; NFI = 0.962; IFI = 0.983. Based on these findings, the full mediation model was retained due to its good fit indices and the absence of nonsignificant paths. In this context, insomnia may be an important link between social media addiction and romantic relationship quality. The results of the full mediation model are presented in Figure 1.

The results indicated that the linkages were statistically significant. As presented in Table 4, social media addiction showed a positive predictive association with psychological inflexibility (β = 0.530, 95% CI [0.421, 0.631], *p* < 0.001). Similarly, social media addiction positively predicted insomnia (β = 0.201, 95% CI [0.058, 0.333], *p* < 0.01), while psychological inflexibility also emerged as a positive predictor of insomnia (β = 0.385, 95% CI [0.260, 0.512], *p* < 0.001). Conversely, insomnia was negatively and significantly associated with romantic relationship quality (β = −0.145, 95% CI [−0.276, −0.017], *p* < 0.05). Likewise, psychological inflexibility was negatively and significantly associated with romantic relationship quality (β = −0.318, 95% CI [−0.444, −0.189], *p* < 0.001). Finally, the serial predictive pathway involving psychological inflexibility and insomnia in the relationship between social media addiction and romantic relationship quality was supported through bootstrapping analysis (β = −0.036, 95% CI [−0.081, −0.007]). The fact that the confidence interval did not include zero indicated that this pathway coefficient was statistically significant.

**Table 4:** Bootstrapping results for the full mediation model.

Paths	Coefficients	95% CI	
		Lower	Upper
Direct			
SMA ➔ PI	0.530	0.421	0.631
SMA ➔ I	0.201	0.058	0.333
PI ➔ I	0.385	0.260	0.512
I ➔ RRQ	−0.145	−0.276	−0.017
PI ➔ RRQ	−0.318	−0.444	−0.189
Indirect			
SMA ➔ PI ➔ I ➔ RRQ	−0.036	−0.081	−0.007

SMA, social media addiction; PI, psychological inflexibility; I, insomnia; RRQ, romantic relationship quality.

### 3.5 Alternative models

In the first alternative model (Model II), Insomnia and Psychological Inflexibility were specified as the exogenous variables, Romantic Relationship Quality as the mediator variable, and Social Media Addiction as the outcome variable. The findings revealed that Model II did not demonstrate adequate fit to the data: χ^2^ (51, *N* = 466) = 264.600, *p* < 0.001; χ^2^/df = 5.188; CFI = 0.903; GFI = 0.910; TLI = 0.874; RMSEA = 0.095, 90% CI [0.084, 0.106]; AIC = 318.600; ECVI = 0.685. In particular, the relatively low TLI value, together with the high CMIN/DF and RMSEA values, indicated that the alternative model was inadequate in explaining the associations among the study variables. These findings suggest that the proposed theoretical model demonstrated relatively better fit to the data compared to Model II.

In the second alternative model (Model III), Insomnia and Psychological Inflexibility were specified as the exogenous variables, Social Media Addiction as the mediator variable, and Romantic Relationship Quality as the outcome variable. The findings indicated that Model III also demonstrated poor fit to the data: χ^2^ (51, *N* = 466) = 215.946, *p* < 0.001; χ^2^/df = 4.234; CFI = 0.925; GFI = 0.927; TLI = 0.903; RMSEA = 0.083, 90% CI [0.072, 0.095]; AIC = 269.946; ECVI = 0.581. More specifically, the elevated Chi-square/df and RMSEA values suggested that this alternative model did not provide adequate fit in explaining the relationships among the study variables. Accordingly, the proposed model was considered to provide a relatively better representation of the observed data than Model III.

When the AIC and ECVI values of all models were compared, the proposed model demonstrated lower values and relatively better fit to the data than the alternative models (Model II and Model III). Taken together, these findings suggest that, within the context of cross-sectional data, the proposed model provides a more appropriate associational framework for explaining the relationships among insomnia, psychological inflexibility, social media addiction, and romantic relationship quality.

## 4 Discussion

The primary aim of this study was to examine the serial predicting roles of psychological inflexibility and insomnia in the relationship between social media addiction and romantic relationship quality. The findings indicated that all hypotheses were supported. The results suggested that social media addiction may be associated with higher levels of psychological inflexibility and insomnia, and that these processes may be reflected in lower romantic relationship quality. In addition, psychological inflexibility and insomnia were found to function as serial mediators in the relationship between social media addiction and romantic relationship quality. Importantly, while the bivariate correlation analysis demonstrated a significant negative relationship between social media addiction and romantic relationship quality, the direct path between these variables became nonsignificant in the final structural equation model (SEM). In Baron and Kenny's (1986) framework, this pattern is not inconsistent; rather, it indicates that the relationship is transmitted through intervening variables. This suggests that the association between social media addiction and romantic relationship quality may operate indirectly through increased psychological inflexibility and subsequent insomnia. These findings indicate that social media addiction and romantic relationship quality may be linked through interconnected psychological and physiological processes. In this respect, the present study contributes to the literature by proposing a comprehensive, process-based framework for understanding how problematic social media use may be reflected in romantic relationship quality. Examining psychological inflexibility and insomnia together within a serial framework provides a more integrative perspective on the relational consequences of social media addiction.

The first finding of the study indicated that social media addiction was negatively associated with romantic relationship quality. This finding is consistent with both earlier and more recent studies indicating that problematic social media use and technology-related behaviors are associated with poorer romantic relationship functioning. Recent evidence suggests that social media addiction and partner phubbing may reduce relationship satisfaction by increasing interpersonal conflict, diminishing emotional responsiveness, and disrupting communication patterns (Karaman and Arslan, 2024; Ni et al., 2025). Similarly, previous studies have shown that social media use may be linked to increased jealousy, insecurity, and conflict in romantic relationships, which may, in turn, be reflected in lower relationship satisfaction (Elphinston and Noller, 2011; Muise et al., 2009). Additionally, social media use has been associated with increased negative interaction patterns in romantic relationships, including partner monitoring, insecurity, and jealousy, which may contribute to lower relationship satisfaction (Fox and Moreland, 2015; Muise et al., 2009). These findings suggest that, while social media may facilitate interpersonal communication, it may also function as a contextual risk factor that weakens trust, intimacy, and commitment dynamics within romantic relationships. Continuous accessibility and visibility through social media may increase comparison, monitoring, and interpretation processes between partners, thereby triggering relational tension. Furthermore, reduced face-to-face interaction and diminished emotional intimacy may also contribute to lower relationship quality. Taken together, these findings suggest that social media addiction should be considered not only in terms of its frequency of use but also in terms of its broader relational consequences.

Another important finding of the study suggests that psychological inflexibility may play a predicting role in the relationship between social media addiction and romantic relationship quality. This finding is consistent with previous research indicating that psychological inflexibility is associated with poorer emotional regulation and interpersonal functioning (Kashdan and Rottenberg, 2010). Social media addiction may contribute to individuals' tendency to avoid unpleasant internal experiences, leading them to engage more frequently in online environments and potentially reinforcing psychological inflexibility. From the perspective of Acceptance and Commitment Therapy, psychological inflexibility is characterized by experiential avoidance and cognitive fusion, which may limit individuals' ability to respond flexibly to challenging emotional experiences (Hayes et al., 2006). Such rigidity may reduce sensitivity to a partner's emotional needs and restrict adaptive interpersonal responses. Consequently, increased psychological inflexibility associated with problematic social media use may undermine relational adjustment and be reflected in lower romantic relationship quality. These findings suggest that psychological inflexibility represents an important psychological process linking problematic social media use with romantic relationship functioning.

The findings further suggest that insomnia may also play a significant predicting role in this relationship. Social media use, particularly during night-time hours, may disrupt individuals' sleep patterns and contribute to insomnia symptoms (Levenson et al., 2016). Increased screen exposure and heightened cognitive arousal may interfere with sleep initiation, sleep duration, and overall sleep quality. Insomnia has been associated with difficulties in emotion regulation, increased irritability, reduced empathy, and poorer interpersonal functioning (Troxel et al., 2007). These emotional and cognitive challenges may contribute to more reactive behaviors, greater conflict, and lower relationship satisfaction within romantic relationships. Insufficient sleep may also impair individuals' ability to accurately interpret social cues and respond sensitively to their partner's emotional needs. Therefore, insomnia may represent an important physiological mechanism through which problematic social media use is reflected in romantic relationship quality.

The main finding of the study suggests that psychological inflexibility and insomnia may operate as serial mediators when considered together. This finding is consistent with the theoretical proposition that problematic social media use may foster psychological inflexibility, which in turn contributes to sleep-related difficulties that are subsequently reflected in poorer romantic relationship quality (Hayes et al., 2006). Rather than representing a simple linear association, these findings support a process-oriented perspective in which psychological and physiological mechanisms jointly explain how problematic social media use may influence romantic relationship functioning. From this perspective, examining psychological inflexibility and insomnia together provides a more comprehensive understanding of the relational consequences of social media addiction. The findings also have important practical implications. Interventions aimed at enhancing psychological flexibility, improving sleep hygiene, and promoting healthier digital habits may help reduce the relational consequences of problematic social media use. Accordingly, preventive programs, psychological counseling interventions, couple-based services, and digital wellbeing initiatives may benefit from simultaneously addressing psychological processes and lifestyle-related behaviors to promote healthier romantic relationships.

### 4.1 Implications

The findings of this study may offer important theoretical and practical implications for understanding the mechanisms underlying the relationship between social media addiction and romantic relationship quality. The results suggest that the associations between social media addiction and romantic relationships may not be limited to a single pathway but may also operate through predictive processes involving psychological and physiological pathways. This perspective indicates that multidimensional and process-based models may provide a more comprehensive understanding of the relational outcomes of social media use. In particular, examining psychological inflexibility and insomnia together may contribute to evaluating these variables—often studied separately in the literature—within a more integrative framework.

From a theoretical perspective, this study may extend the role of psychological inflexibility within the context of interpersonal relationships from the standpoint of Acceptance and Commitment Therapy. Psychological inflexibility may not be limited to individual wellbeing but also be reflected in romantic relationship quality. In addition, considering psychological inflexibility and insomnia together may help to illuminate the interaction between cognitive-emotional processes and physiological processes. In this context, approaches based on multi-step and serial mediation models may provide a novel perspective for explaining the relational outcomes of social media addiction.

From a research perspective, these findings may contribute to the development of new research questions aimed at examining the relationships among social media addiction, psychological inflexibility, and sleep-related processes across different samples and research designs. In particular, experimental and longitudinal studies may help to provide a clearer understanding of the potential causal relationships among these variables. In addition, cross-cultural comparisons and studies conducted across different age groups may be valuable for examining the generalizability of these findings.

From a practitioner perspective, the findings may highlight the importance of holistic approaches in psychological counseling and clinical intervention processes. Rather than focusing solely on reducing social media use when addressing difficulties in romantic relationships, interventions aimed at enhancing individuals' psychological flexibility may be considered. Approaches grounded in Acceptance and Commitment Therapy may support individuals in developing a more flexible relationship with their internal experiences, which may contribute to improved relational adjustment. In addition, psychoeducation on sleep hygiene and behavioral regulation strategies may be considered as functional intervention areas for addressing the intervening pathways predicted by social media use. Addressing these components together may contribute to the development of more comprehensive interventions targeting both psychological processes and lifestyle-related habits.

Finally, from a policy perspective, these findings may inform the development of digital wellbeing and mental health policies. In particular, promoting awareness programs targeting social media use among young adults and university students, encouraging healthy digital habits, and supporting education aimed at maintaining healthy sleep patterns may be important. School- and university-based preventive programs may contribute to enhancing individuals' psychological flexibility and fostering healthier relationships with digital environments. In this regard, considering that social media addiction may have not only individual but also relational implications, the development of multidimensional and preventive mental health policies may be recommended. Taken together, these findings suggest that interventions targeting problematic social media use should not focus solely on reducing screen time but should also strengthen psychological flexibility, promote healthy sleep behaviors, and foster digital wellbeing to support healthier romantic relationships.

### 4.2 Limitations and future research

Although the findings of this study may provide important contributions, they should be considered within the context of several limitations. First, the cross-sectional design of the study may limit causal interpretations of the relationships among variables. Therefore, future longitudinal or experimental studies may contribute to a clearer examination of the potential causal relationships among social media addiction, psychological inflexibility, and insomnia. Second, the reliance on self-report measures may introduce potential biases, such as social desirability and recall bias. In this regard, future research may benefit from incorporating behavioral indicators, partner reports, or objective measures (e.g., digital usage data or sleep tracking) to provide more robust support for the findings.

Third, the use of a specific sample may limit the generalizability of the findings. Studies conducted across different age groups, cultural contexts, and relationship types may be valuable for examining the validity of the proposed model in diverse populations. Cross-cultural comparisons may contribute to understanding how the relational outcomes of social media use vary across contexts.

Fourth, this study focused only on psychological inflexibility and insomnia as explanatory factors. However, the relationship between social media addiction and romantic relationship quality is likely to be shaped by a broader range of psychological, relational, and behavioral processes. Future studies may benefit from incorporating variables such as relationship duration, partner social media use, partner phubbing, attachment styles, emotional regulation, fear of missing out (FoMO), depressive and anxiety symptoms, romantic jealousy, rumination, digital jealousy, and other addictive behaviors to provide a more comprehensive understanding of this relationship. In addition, future research may benefit from employing dyadic, longitudinal, experimental, and cross-cultural designs to clarify the temporal and interpersonal mechanisms underlying these associations. Considering these factors simultaneously may provide a more nuanced understanding of how problematic social media use influences romantic relationship quality across different relational contexts.

## 5 Conclusion

This study sheds light on the underlying mechanisms of the relationship between social media addiction and romantic relationship quality by examining psychological inflexibility and insomnia. The findings suggest that social media addiction may be associated with higher levels of psychological inflexibility and insomnia and that these processes may be reflected in lower romantic relationship quality. In addition, psychological inflexibility and insomnia function as serial mediators in this relationship. These results indicate that the relational outcomes of social media addiction may not be limited to behavioral processes but also involve cognitive-emotional and physiological mechanisms. In this regard, adopting holistic and multidimensional approaches may be important for understanding the relational implications of social media use. Overall, this study proposes a model that may contribute to the literature, and the findings could provide guidance for future research and intervention efforts.

## Data Availability

The raw data supporting the conclusions of this article will be made available by the authors, without undue reservation.

## References

[B1] Anderson J. C. Gerbing D. W. (1988). Structural equation modeling in practice: a review and recommended two-step approach. Psychol. Bull. 103, 411–423. doi: 10.1037/0033-2909.103.3.411

[B2] Andreassen C. S. (2015). Online social network site addiction: a comprehensive review. Curr. Addict. Rep. 2, 175–184. doi: 10.1007/s40429-015-0056-9

[B3] Andreassen C. S. Billieux J. Griffiths M. D. Kuss D. J. Demetrovics Z. Mazzoni E. . (2016). The relationship between addictive use of social media and video games and symptoms of psychiatric disorders: a large-scale cross-sectional study. Psychol. Addict. Behav. 30, 252–262. doi: 10.1037/adb0000160 26999354

[B4] Baron R. M. Kenny D. A. (1986). The moderator–mediator variable distinction in social psychological research: conceptual, strategic, and statistical considerations. J. Pers. Soc. Psychol. 51, 1173–1182. doi: 10.1037/0022-3514.51.6.1173 3806354

[B5] Bond F. W. Hayes S. C. Baer R. A. Carpenter K. M. Guenole N. Orcutt H. K. . (2011). Preliminary psychometric properties of the acceptance and action questionnaire–II: a revised measure of psychological inflexibility and experiential avoidance. Behav. Ther. 42, 676–688. doi: 10.1016/j.beth.2011.03.007 22035996

[B6] Chang A. M. Aeschbach D. Duffy J. F. Czeisler C. A. (2015). Evening use of light-emitting eReaders negatively affects sleep, circadian timing, and next-morning alertness. Proc. Natl. Acad. Sci. USA. 112, 1232–1237. doi: 10.1073/pnas.1418490112 25535358 PMC4313820

[B7] Demirci I. (2019). The adaptation of the Bergen social media addiction scale to Turkish and its evaluation of relationship with depression and anxiety symptoms. Anatolian J. Psychiat. 20, 15–22. doi: 10.5455/apd.41585

[B8] Elbi H. Batum M. Öztürk E. Ö. Vatansever Balcan M. Kisabay Ak A. Yilmaz H. . (2024). Adaptation into Turkish and psychometric properties of Athens insomnia scale. Arch. Neuropsychiatry 61, 154–159. doi: 10.1037/t94730-000 38868854 PMC11165601

[B9] Elphinston R. A. Noller P. (2011). Time to face it! Facebook intrusion and the implications for romantic jealousy and relationship satisfaction. Cyberpsychol. Behav. Soc. Netw. 14, 631–635. doi: 10.1089/cyber.2010.0318 21548798

[B10] Field A. (2016). Discovering Statistics Using IBM SPSS Statistics, North American Edn. London: SAGE Publications.

[B11] Fletcher G. J. O. Simpson J. A. Thomas G. (2000). The measurement of perceived relationship quality components: a confirmatory factor analytic approach. Pers. Soc. Psychol. Bull. 26, 340–354. doi: 10.1177/0146167200265007

[B12] Fornell C. Larcker D. F. (1981). Evaluating structural equation models with unobservable variables and measurement error. J. Mark. Res. 18, 39–50. doi: 10.1177/002224378101800104

[B13] Fox J. Moreland J. J. (2015). The dark side of social networking sites: an exploration of the relational and psychological stressors associated with Facebook use and affordances. Comput. Human Behav. 45, 168–176. doi: 10.1016/j.chb.2014.11.083

[B14] Gordon A. M. Chen S. (2014). The role of sleep in interpersonal conflict: do sleepless nights mean worse fights? Soc. Psychol. Personal. Sci. 5, 168–175. doi: 10.1177/1948550613488952

[B15] Hair J. F. Black W. C. Babin B. J. Anderson R. E. (2019). Multivariate Data Analysis, 8th Edn. New York, NY: Cengage Learning.

[B16] Hayes A. F. (2018). Introduction to Mediation, Moderation, and Conditional Process Analysis: A Regression-Based Approach, 2nd Edn. New York, NY: The Guilford Press.

[B17] Hayes S. C. Luoma J. B. Bond F. W. Masuda A. Lillis J. (2006). Acceptance and commitment therapy: model, processes and outcomes. Behav. Res. Ther. 44, 1–25. doi: 10.1016/j.brat.2005.06.006 16300724

[B18] Henseler J. Ringle C. M. Sarstedt M. (2015). A new criterion for assessing discriminant validity in variance-based structural equation modeling. J. Acad. Mark. Sci. 43, 115–135. doi: 10.1007/s11747-014-0403-8

[B19] Hoyle R. H. Panter A. T. (1995). “Writing about structural equation models,” in Structural Equation Modeling: Concepts, Issues, and Applications, ed. R. H. Hoyle (Sage), 158–176.

[B20] Huang C. (2022). A meta-analysis of the problematic social media use and mental health. Int. J. Soc. Psychiatry 68, 12–33. doi: 10.1177/0020764020978434 33295241

[B21] Karaman M. A. Arslan G. (2024). The mediating role of social media addiction and phubbing in the relationship between basic psychological needs and relationship satisfaction. Curr. Psychol, 15:1291638. doi: 10.3389/fpsyg.2024.1291638 38586290 PMC10995373

[B22] Kashdan T. B. Rottenberg J. (2010). Psychological flexibility as a fundamental aspect of health. Clin. Psychol. Rev. 30, 865–878. doi: 10.1016/j.cpr.2010.03.001 21151705 PMC2998793

[B23] Kline R. B. (2023). Principles and Practice of Structural Equation Modeling, 5th Edn. New York, NY: The Guilford Press.

[B24] Kuss D. J. Griffiths M. D. (2017). Social networking sites and addiction: ten lessons learned. Int. J. Environ. Res. Public Health 14:311. doi: 10.3390/ijerph14030311 28304359 PMC5369147

[B25] Levenson J. C. Shensa A. Sidani J. E. Colditz J. B. Primack B. A. (2016). The association between social media use and sleep disturbance among young adults. Prev. Med. 85, 36–41. doi: 10.1016/j.ypmed.2016.01.001 26791323 PMC4857587

[B26] Little T. D. Cunningham W. A. Shahar G. Widaman K. F. (2002). To parcel or not to parcel: exploring the question, weighing the merits. Struct. Equ. Model. 9, 151–173. doi: 10.1207/S15328007SEM0902_1

[B27] Little T. D. Rhemtulla M. Gibson K. Schoemann A. M. (2013). Why the items versus parcels controversy needn't be one. Psychol. Methods 18, 285–300. doi: 10.1037/a0033266 23834418 PMC3909043

[B28] Muise A. Christofides E. Desmarais S. (2009). More information than you ever wanted: does Facebook bring out the green-eyed monster of jealousy? J. Cyber. Rehabil. 12, 441–444. doi: 10.1089/cpb.2008.0263 19366318

[B29] Nasser-Abu Alhija F. Wisenbaker J. (2006). A Monte Carlo study investigating the impact of item parceling strategies on parameter estimates and their standard errors in CFA. Struct. Equ. Model. 13, 204–228. doi: 10.1207/s15328007sem1302_3

[B30] Ni X. Ahrari S. Zaremohzzabieh Z. Zarean M. Roslan S. (2025). The relationship between partner phubbing and relationship satisfaction: a systematic review and meta-analysis. Front. Psychol. 16:1561159. doi: 10.3389/fpsyg.2025.1561159 40432797 PMC12106345

[B31] Oltra J. Amigo Vázquez I. Secades-Villa R. (2024). How does smoking tobacco affect choosing a stable partner? Adicciones 36, 63–68. doi: 10.20882/adicciones.1709 36200226

[B32] Roberts J. A. David M. E. (2016). My life has become a major distraction from my cell phone: partner phubbing and relationship satisfaction. Comput. Human Behav. 54, 134–141. doi: 10.1016/j.chb.2015.07.058

[B33] Sagkal A. S. Özdemir Y. (2018). Turkish adaptation of perceived romantic relationship quality scale (PRRQS): validity and reliability study. Mehmet Akif Ersoy Univ. J. Educ. Fac. 46, 22–40. doi: 10.21764/maeuefd.329888

[B34] Soldatos C. R. Dikeos D. G. Paparrigopoulos T. J. (2000). Athens insomnia scale: validation of an instrument based on ICD-10 criteria. J. Psychosom. Res. 48, 555–560. doi: 10.1016/S0022-3999(00)00095-7 11033374

[B35] Tabachnick B. G. Fidell L. S. (2013). Using Multivariate Statistics, 6th Edn. Boston, MA: Pearson.

[B36] Troxel W. M. Robles T. F. Hall M. Buysse D. J. (2007). Marital quality and the marital bed: examining the covariation between relationship quality and sleep. Sleep Med. Rev. 11, 389–404. doi: 10.1016/j.smrv.2007.05.002 17854738 PMC2644899

[B37] Yang C. Nay S. Hoyle R. H. (2010). Three approaches to using lengthy ordinal scales in structural equation models: parceling, latent scoring, and shortening scales. Appl. Psychol. Meas. 34, 122–142. doi: 10.1177/0146621609338592 20514149 PMC2877522

[B38] Yavuz F. Ulusoy S. Iskin M. Esen F. B. Burhan H. S. Karadere M. E. . (2016). Turkish version of acceptance and action questionnaire-II (AAQ-II): a reliability and validity analysis in clinical and non-clinical samples. Bull. Clin. Psychopharmacol. 26, 397–408. doi: 10.5455/bcp.20160223124107

